# A Rare Case of Complete Myxoma Detachment Leading to Abdominal Aortic Occlusion and Secondary Visceral Necrosis: A Case Description and an Analysis of the Literature

**DOI:** 10.3390/jcm14186526

**Published:** 2025-09-17

**Authors:** Xu Hu, Wenzhao Zhang, Jianqun Yu

**Affiliations:** 1 Department of Emergency, West China Hospital, Sichuan University, 37 Guo Xue Alley, Chengdu 610041, China; 2 Department of Radiology, West China Hospital, Sichuan University, 37 Guo Xue Alley, Chengdu 610041, China

**Keywords:** cardiac myxoma, computed tomography angiography, surgery

## Abstract

Complete detachment of a cardiac myxoma represents an exceptionally rare but potentially catastrophic complication. This case report describes a young female patient who developed acute abdominal pain following vigorous physical exertion, with rapid progression to visceral ischemia and bilateral lower limb ischemia within an extremely short timeframe. Comprehensive diagnostic imaging and postoperative pathological examination confirmed this as a remarkably rare case of complete cardiac myxoma detachment. This condition has been reported in only a handful of cases in the existing medical literature.

## 1. Introduction

A cardiac myxoma is a common benign cardiac tumor, yet its exact etiology remains unclear [1]. The clinical manifestations of myxomas vary widely, ranging from asymptomatic cases to sudden death [2]. Due to its gelatinous and friable structure, a myxoma carries a high risk of embolization, which may lead to acute vascular events in the pulmonary, systemic, or even coronary circulation [3,4]. Embolic complications can severely compromise patient survival and quality of life. However, the clinical factors predisposing myxoma patients to embolic events remain poorly understood. This article presents a rare case of a young female patient who suffered complete myxoma detachment, resulting in abdominal aortic occlusion and subsequent multivisceral ischemic infarction. A literature review is also provided to discuss relevant clinical implications.

## 2. Case Report

A 27-year-old female in early pregnancy (<4 weeks of gestation) presented to the emergency department with a 3 h history of persistent lower abdominal pain and bilateral lower extremity numbness following physical exertion (running). The pain radiated to the lumbar region and was accompanied by progressive lower limb weakness and paresthesia. An initial evaluation at an outside hospital, including abdominal ultrasonography, revealed no significant abnormalities. She was subsequently transferred to the Emergency Department of West China Hospital, Sichuan University, for further management. Physical examination revealed the following: The bilateral lower extremities were cold with diminished sensation. Vascular observations indicated absent dorsalis pedis pulses bilaterally. A venous blood test showed elevated D-dimer levels, 2.17 mg/L FEU (reference value < 0.55 mg/L FEU). The absolute value of neutrophils increased to 7.73 × 10^9^/L (reference value 1.8–6.3 × 10^9^/L), and the percentage of neutrophils increased to 89.4% (reference value 40–75%). Lactate, <1.00 mmol/L (reference value 0.7–2.1). When the patient was admitted, her blood pressure was 116/79.

**Figure 1:**
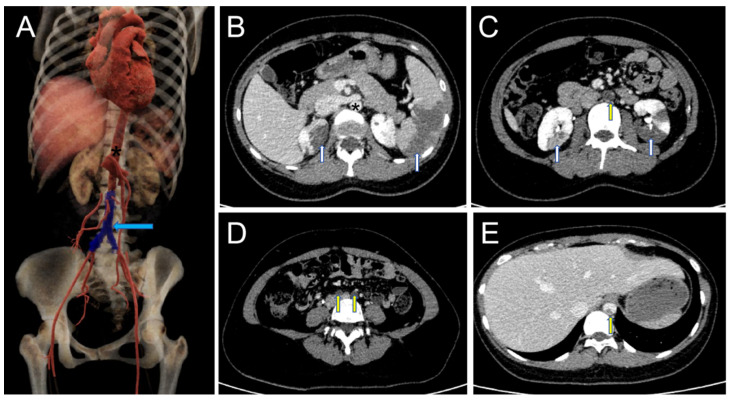
(**A**). Volume rendering (VR) showing the occlusion of the renal lower abdominal aorta and bilateral common iliac arteries, thrombus filling in the lumen, no contrast agent imaging (blue arrow), and the abdominal aorta (black *). (**B**). Showing multiple ischemic infarctions in the kidneys and the spleen (white arrows) and the abdominal aorta (black *). (**C**). Showing the occlusion of the abdominal aorta without contrast agent filling (yellow arrow) and multiple ischemic infarctions in the filled kidneys (white arrow). (**D**). Showing the bilateral common iliac artery occlusion and no contrast agent filling (yellow arrow). (**E**). Showing a slight filling defect in the descending thoracic aorta lumen (yellow arrow).

**Figure 2:**
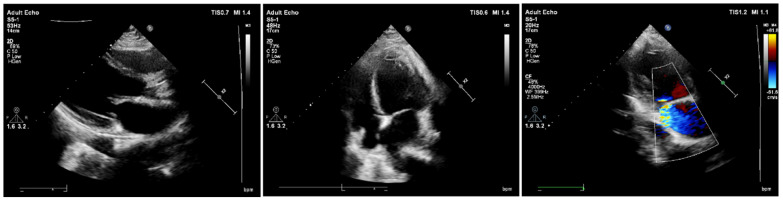
TTE showing no abnormalities in the patient’s heart.

Urgent chest and abdominal CTA examination (computed tomography angiography) was performed, and CTA vascular enhancement images showed multiple filling defects in the renal lower abdominal aorta, bilateral common iliac arteries, bilateral internal and external iliac arteries, and the distal lumen of the left renal artery. The enhanced scan showed mild to moderate enhancement. However, no contrast agent was found in the renal lower abdominal aorta or bilateral common iliac artery lumens, and the lumens were completely occluded for a length of about 11.4 cm. Multiple patchy enhancement reduction areas were observed in both kidneys and the spleen, suggesting multiple ischemic infarctions. Another nodular filling defect with a size of approximately 0.8 × 0.5 cm was observed in the descending thoracic aorta (Figure 1). The patient subsequently underwent transthoracic echocardiography (TTE) examination, which showed no significant abnormalities in the heart (Figure 2).

**Figure 3:**
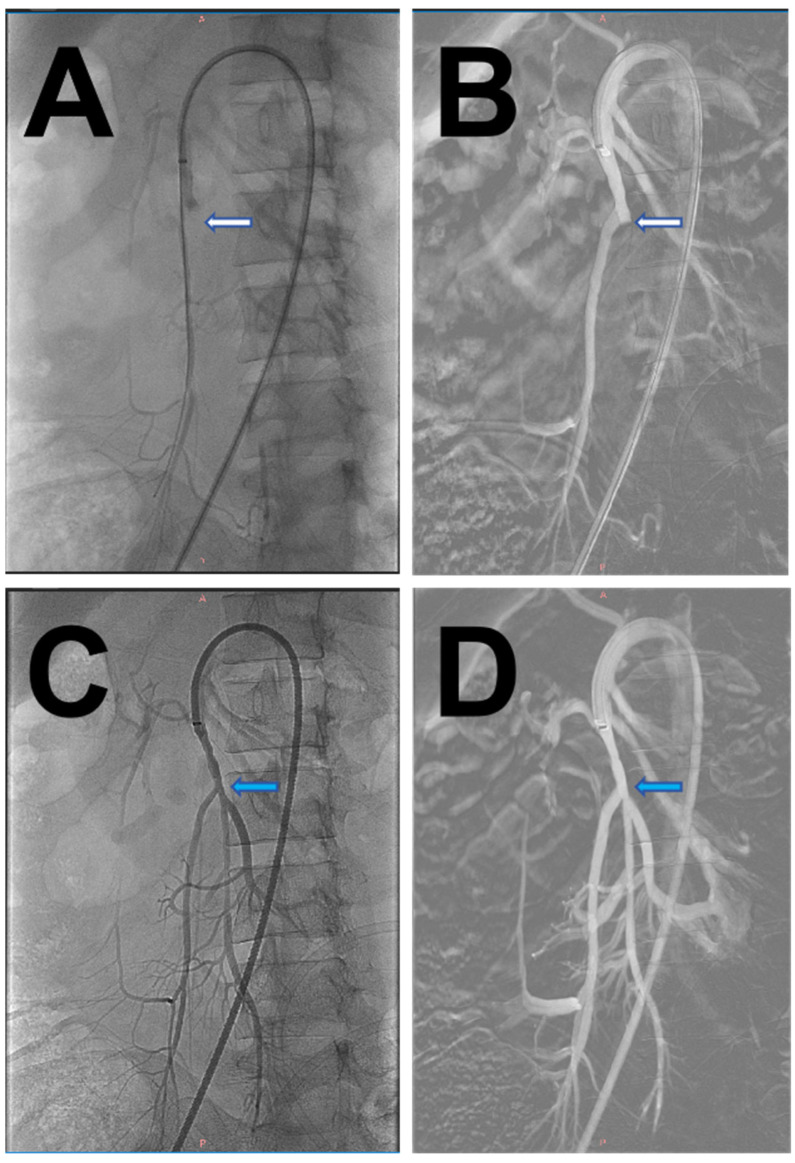
Intraoperative Digital Subtraction Angiography (DSA) examination images of the patient. (**A**,**B**). The patient had an occlusion of the renal lower abdominal aorta and bilateral iliac arteries (white arrows). (**C**,**D**) After interventional thrombectomy, the patient’s abdominal aorta and iliac artery resumed reperfusion (blue arrow).

**Figure 4:**
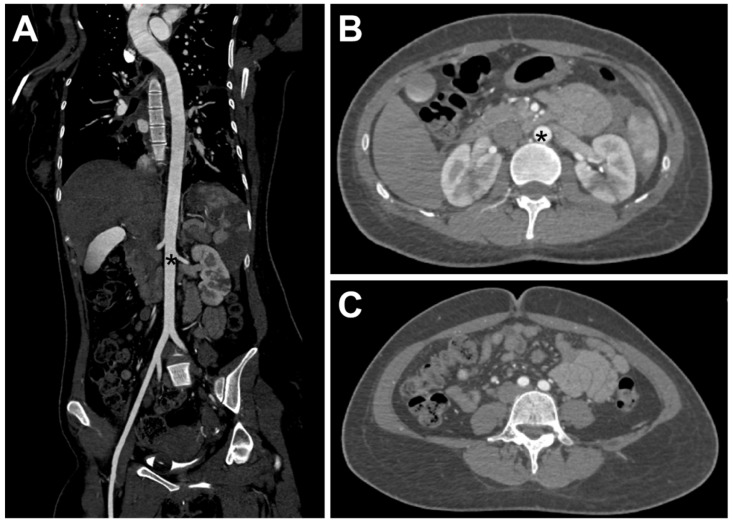
(**A**–**C**). After interventional thrombectomy, the patient’s abdominal aorta and bilateral iliac arteries resumed reperfusion, with good contrast agent filling and no filling defects observed. The abdominal aorta is shown (black *).

The patient agreed to undergo surgical treatment and terminate the pregnancy. The patient underwent emergency bilateral femoral artery thrombectomy with aortoiliac angiography, which revealed complete occlusion of the distal abdominal aorta below the renal arteries, bilateral common iliac arteries, and bilateral internal iliac arteries. The diameter of the bilateral common femoral arteries was about 5 mm, and no pulsation was palpable. The common femoral artery was cut open using V18 guidewire assistance, and two 5F Fogarty balloon catheters were advanced approximately 40 cm into the abdominal aorta using bilateral femoral approaches. Three sequential passes retrieved multiple large, semi-transparent emboli, resulting in immediate restoration of robust distal back-bleeding and proximal forward flow. Post-thrombectomy angiography demonstrated successful recanalization of the abdominal aorta, bilateral iliac arteries, and common femoral arteries (Figure 3), with palpable dorsalis pedis pulses bilaterally. Postoperative CTA confirmed complete resolution of the previous filling defects, showing normal perfusion of the abdominal aorta and bilateral common iliac arteries without residual thrombi or emboli (Figure 4).

**Figure 5:**
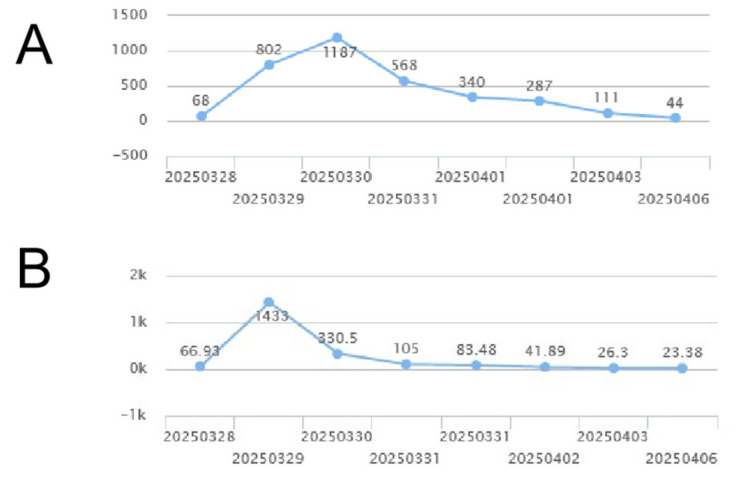
The trajectories of creatine kinase (**A**) and creatinine (**B**).

Postoperative subcutaneous injection of heparin, 0.5 mL each time, twice a day for a total of three days. The trajectories of creatinine, creatine kinase, and creatinine before and after surgery are shown in Figure 5.

**Figure 6:**
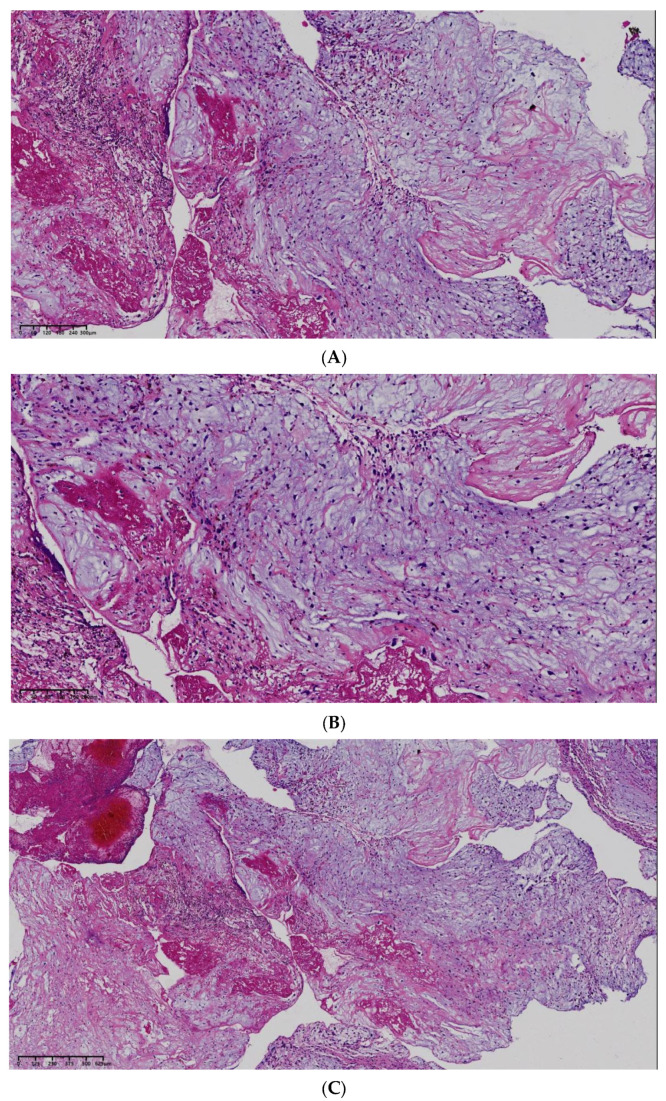
Pathological results: Hematoxylin and eosin staining ((**A**–**C**), 4× magnification, 6× magnification, 10× magnification) suggesting myxoma.

Pathological examination revealed that the embolus had a grayish-white, solid, and moderately firm cut surface. Microscopically, an abundant myxoid matrix was observed with scattered or clustered medium-sized cells. Immunohistochemical staining showed positivity for CR (+), WT1 (−), CD34 (+), CD31 (−), SMA (−), CK (Pan) (−), EMA (−), S100 (−), Desmin (−), p63 (+, Minority), MDM2 (+/−), INI1 (not missing), CDK4 (−), and Ki-67 (MIB-1) (+, individual). Based on the morphological features and immunohistochemical profile, the diagnosis of myxoma was established (Figure 6).

**Figure 7:**
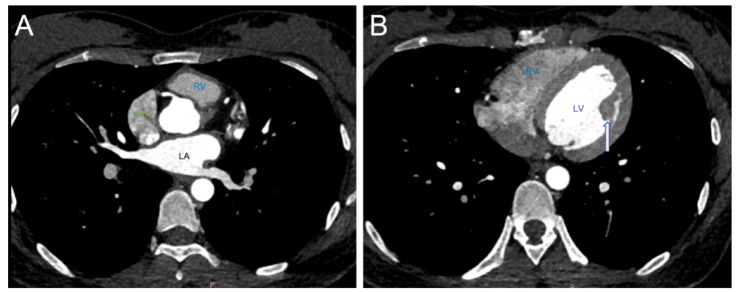
The patient underwent a follow-up cardiac CT scan (**A**,**B**), which found no abnormalities in either atrium. The left atrium (LA), right atrium (RA), and right ventricle (RV) were not found. (**B**). A large papillary muscle in the left ventricle (white arrow), right ventricle (RV), and left ventricle (LV).

The patient recovered well postoperatively and was discharged on postoperative day 7 without reporting any significant discomfort. Follow-up contrast-enhanced cardiac CT at 15 days postoperation demonstrated no cardiac enlargement and a large papillary muscle in the left ventricle (Figure 7). There is a small filling defect in the descending aorta of the original chest, which was not shown this time. The contrast agent in the lumen is well filled. Subsequent transthoracic echocardiography (TTE) performed 4 months after the procedure continued to show no apparent abnormalities.

**Figure 8:**
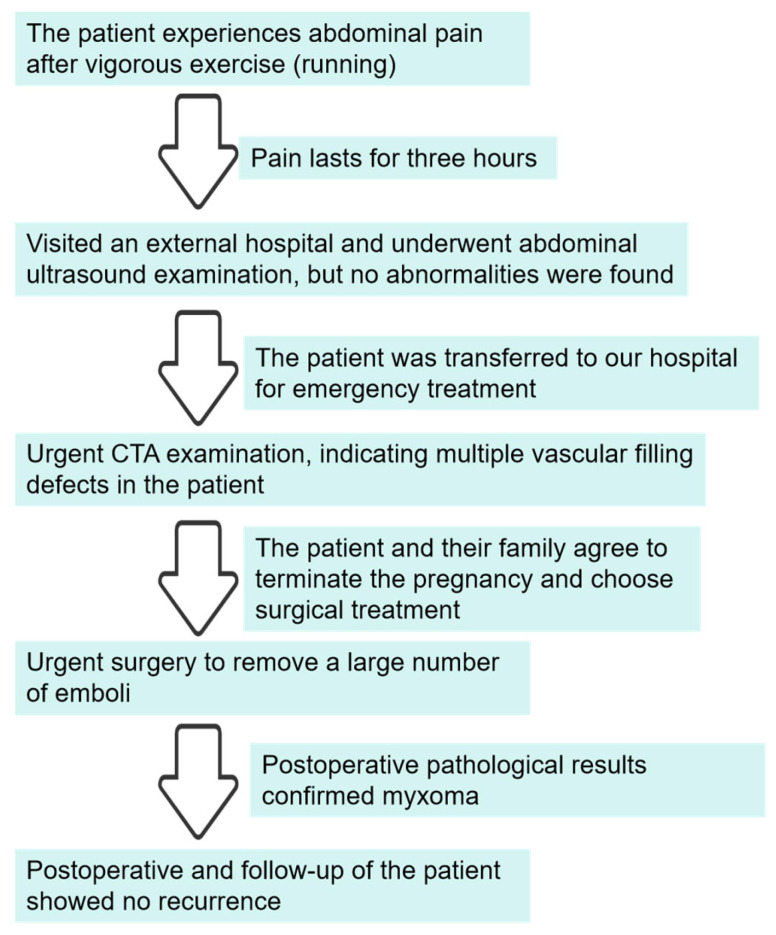
The patient treatment process.

The patient treatment process is shown in the timeline figure, illustrated in Figure 8.

Based on the comprehensive evaluation of all clinical findings, this case represents a rare occurrence of complete myxoma detachment leading to extensive embolism in the infrarenal abdominal aorta and iliac arteries, resulting in multifocal ischemic infarctions of the spleen and kidneys, along with acute lower limb ischemia.

## 3. Discussion and Conclusions

Primary cardiac tumors are exceptionally rare, with an autopsy-reported prevalence of 0.017–0.19% [5]. Cardiac myxomas, the most common primary cardiac tumors, originate from primitive multipotent mesenchymal stem cells in the endocardial region of the fossa ovalis and are predominantly sporadic [5]. These tumors demonstrate a female predominance (aged 30–60 years), with 80–90% located in the left atrium and most remaining cases in the right atrium [6,7]. Myxomas can present with various clinical symptoms, mainly including difficulty breathing and palpitations. This may be because the tumors occupy the heart cavity and are pushed by blood, causing changes in the hemodynamics of the heart cavity, which, in turn, can lead to arrhythmia and changes in heart function. About 93.9% of patients show cardiac symptoms; however, some may also be asymptomatic, as shown in Table 1 [8]. Embolic complications occur in up to 30% of cases [7,8,9], with cerebral embolism (27–55%) manifesting as ischemic stroke, transient ischemic attack, or retinal artery occlusion being the most common [10]. Previously, it was reported that a patient experienced extensive abdominal aortic embolism without any prior symptoms [11,12].

Complete myxoma detachment causing aortic occlusion remains extraordinarily rare [13,14]. According to reports, the clinical features of complete aortic occlusion caused by myxomas are similar to those of aortic dissection [15]. Eriksen et al. [16] described a patient with a left atrial myxoma who first experienced cerebral embolism, which occurred 15 h after the bifurcation of the iliac artery. The patient passed away three days after surgery. During the autopsy, no residual mass was found in the heart, but there was a rough surface area in the left atrium, which may have been the site of myxoma attachment. Kulkarni et al. [17] described a patient with a left atrial myxoma, whose tumor completely detached and subsequently led to a saddle embolism. The patient underwent surgery to remove the embolus but passed away shortly after the operation. During the autopsy, no residual tumor was found in the heart, but a recently formed thrombus was discovered in the posterior wall of the left atrium, which may have been the location of the myxoma attachment.

Transthoracic echocardiography (TTE) is currently one of the preferred non-invasive examination methods for cardiac myxomas. Its advantages include high detection accuracy and the ability to observe the size, shape, location, and internal echoes of myxomas, which is of great significance for localizing myxomas and developing surgical plans [18].

Multi-row spiral CTA examination can comprehensively scan the chest and abdominal blood vessels and the heart in just a few seconds. It can clearly display the size, morphology, and origin of tumors in the large blood vessels of the heart and clarify the density characteristics of tumors, such as soft tissue density, low density, or calcification density. Tumors in the heart cavity can be displayed in relation to the overall heart cavity. Cardiac mucinous tumors can be detected by CT and are often connected to the heart wall through the pedicle. Combined with multi-planar reconstruction and volume rendering techniques, tumor pedicle attachment points (commonly near the oval fossa) can be presented in three dimensions, which is crucial for surgical planning. However, the visualization of tumor pedicles may not be as sensitive as transesophageal echocardiography (TEE).

The patient in this case presented to our emergency department. The emergency physician at our institution noted several key clinical features: (1) a history of vigorous physical exertion preceding the onset of thoracolumbar pain, (2) early pregnancy status, conferring a hypercoagulable state, and (3) mildly elevated D-dimer levels. The patient subsequently developed rapidly progressive neurological deficits in the lower extremities, including decreased motor strength, cutaneous hypothermia, and bilateral paresthesia—a clinical constellation concerning for aortic dissection. Given the broad differential diagnosis that included acute myelitis and thromboembolic disease, emergent thoracoabdominal computed tomography angiography (CTA) was promptly obtained for definitive evaluation.

Computed tomography angiography (CTA) revealed extensive embolic occlusion involving the infrarenal abdominal aorta and bilateral common iliac arteries, with complete filling of the vascular lumen (Figure 1C,D). The emboli demonstrated heterogeneous mild-to-moderate enhancement on contrast-enhanced scanning. Enhancement that may be seen in tumor emboli due to intralesional microvasculature [19]. Considering the patient’s age, gender, and clinical manifestations, we suspected that the aortic embolism was caused by the detachment of a myxoma rather than being a conventional thrombotic vascular occlusion. However, in contrast to typical cases of myxoma detachment, both the preoperative transthoracic echocardiography (TTE) and postoperative cardiac CT follow-up examinations in this patient showed no abnormalities in the heart. The preoperative CTA did reveal minor filling defects in the descending thoracic aorta (Figure 1E), which we speculate may represent residual fragments from the myxoma detachment process. The myxoma diagnosis was subsequently confirmed by postoperative pathological biopsy (Figure 5). Based on these findings, we conclude that this patient represents an extremely rare case of complete cardiac myxoma detachment. The gelatinous structural characteristics of myxomas render conventional thrombolytic therapy ineffective, making surgical intervention the only viable treatment option. In this case, the extensive occlusion of major abdominal vessels precipitated rapid-onset ischemic necrosis in both the abdominal organs and lower extremities within an extremely short timeframe. Without prompt intervention, the consequences could have been catastrophic. The successful treatment of this patient was attributed to three critical factors: the emergency physician’s accurate clinical judgment, the appropriate selection of diagnostic imaging modalities, and the timely performance of surgical thrombectomy.

## 4. Conclusions

This case report demonstrates that when patients experience acute arterial embolism symptoms, appropriate imaging examinations should be selected. Early diagnosis using these modalities can significantly reduce the incidence of arterial occlusion events, enhance treatment efficacy, and improve outcomes related to reperfusion complications. CTA serves as an important auxiliary diagnostic tool for evaluating systemic vascular pathology, offering advantages such as rapid acquisition time, extensive anatomical coverage, and unique value in assessing anatomical details, conducting preoperative planning, and evaluating complications. Due to the emergent nature of the presentation, the patient in this case did not complete transthoracic echocardiography (TTE) in a timely manner during preoperative examination. TEE should be considered when feasible. By combining TTE, the accuracy of diagnosis can be improved. Ultimately, definitive diagnosis still relies on pathological biopsy confirmation.

A cardiac myxoma should be suspected from the imaging characteristics of the embolus, warranting prompt surgical intervention, even in the absence of abnormal findings on transthoracic echocardiography.

## Figures and Tables

**Table 1:** Visualization of prior reports.

Feature				
Age	mean age 55 years (range 22–79 years)			
Sex	males (24.5%)		females (75.5%)	
Primary site	left atrium (87.7%)	mitral valve (6.1%)	right atrium (4.1%)	left and right atria (2.0%)
Occlusion site	left ventricle (40.8%)	mitral stenosis (10.2%)	threatened left ventricular outflow tract obstruction(2%)	
Clinical symptoms	Cardiac signs appeared (93.9%)		Preoperative embolic events had occurred (26.5%)	
outcomes	patients remained without cardiac symptoms (81.1%)	early mortality rate (2.0%)	late mortality rate (6.1%)	actuarial survival rate (0.74)

## Data Availability

The original contributions presented in this study are included in the article. Further inquiries can be directed to the corresponding author.
